# Environmental gradients shape viral-host dynamics in the Pearl River estuary

**DOI:** 10.1093/ismeco/ycaf164

**Published:** 2025-09-17

**Authors:** Ruixian Sun, Wenqian Xu, Yangbing Xu, Zhimeng Xu, Yehui Tan, Jiying Li, Hongbin Liu, Charmaine C M Yung

**Affiliations:** Department of Ocean Science, The Hong Kong University of Science and Technology, Hong Kong SAR, China; Department of Ocean Science, The Hong Kong University of Science and Technology, Hong Kong SAR, China; Department of Ocean Science, The Hong Kong University of Science and Technology, Hong Kong SAR, China; Department of Ocean Science, The Hong Kong University of Science and Technology, Hong Kong SAR, China; Haide College, Ocean University of China, Qingdao, 266100, China; Key Laboratory of Tropical Marine Bio-resources and Ecology, South China Sea Institute of Oceanology Chinese Academy of Sciences, Guangzhou, 510399, China; Department of Ocean Science, The Hong Kong University of Science and Technology, Hong Kong SAR, China; Department of Ocean Science, The Hong Kong University of Science and Technology, Hong Kong SAR, China; Department of Ocean Science, The Hong Kong University of Science and Technology, Hong Kong SAR, China; Southern Marine Science and Engineering Guangdong Laboratory (Guangzhou), The Hong Kong University of Science and Technology, Hong Kong SAR, China

**Keywords:** virome, Pearl River estuary, virus-host interaction, environmental microbiome, environmental gradients

## Abstract

Marine viruses play critical roles in shaping microbial communities and driving biogeochemical cycles, yet their dynamics in estuarine systems are not well characterized. Here, we conducted a comprehensive metagenomic analysis of viral communities and virus-host interactions across the Pearl River estuary, a dynamic subtropical estuary in southern China. Using 24 metagenomic libraries from eight sampling sites, we identified 29,952 viral populations, with Uroviricota and potential Uroviricota accounted for 80.48% of taxa, underscoring their ecological importance. A key finding of our integrated analysis is the unexpectedly high abundance of nucleocytoplasmic large DNA viruses in offshore waters, which suggests a more significant role for eukaryotic viruses in coastal ecosystems than previously acknowledged and correlates with elevated levels of their eukaryotic hosts. Environmental variables, particularly salinity and nutrient availability, emerged as key drivers of viral and host distribution patterns. By linking environmental gradients to distinct community “envirotypes” and their underlying genomic features, we revealed novel virus-host interactions and highlighted the impact of environmental gradients on microbial ecology. Additionally, viral auxiliary metabolic genes linked to phosphorus and nitrogen metabolism suggest critical roles in modulating host metabolic pathways and influencing nutrient cycling. Our findings demonstrate how spatial heterogeneity and environmental gradients shape viral and microbial ecology in estuarine ecosystems. Our findings provide a holistic, multi-domain view of microbial and viral ecology, demonstrating how integrating prokaryotic, eukaryotic, and viral community analyses offers a more complete understanding of ecosystem function in these critical transition zones.

## Introduction

Estuaries are critical ecological transition zones that connect freshwater and marine environments, facilitating the exchange of dissolved and particulate matter while supporting diverse and dynamic ecosystems [1]. These dynamic systems are characterized by steep environmental gradients, including changes in salinity, nutrient availability, and turbidity, which create unique ecological niches for microbial communities. Microbial communities, comprising prokaryotes, protists, and viruses, drive key biogeochemical cycles in estuaries and respond rapidly to environmental perturbations [2, 3]. Among them, viruses play a pivotal role in shaping microbial dynamics through host mortality, horizontal gene transfer, and metabolic reprogramming, directly influencing nutrient turnover and ecosystem stability [4, 5]. Despite their critical ecological functions, our understanding of viral diversity, distribution, and interactions with microbial hosts in estuarine systems remains limited.

The Pearl River estuary (PRE), a major subtropical estuary in southern China, provides an ideal natural laboratory for studying microbial and viral ecology. Spanning approximately 2600 km^2^ along the northern boundary of the South China Sea, the PRE is shaped by the confluence of inputs from the Pearl River, South China Sea, and coastal currents [6]. This hydrodynamic complexity generates strong environmental gradients in salinity and nutrient concentrations, which significantly influence microbial and viral community composition [7–10]. Additionally, the PRE is heavily impacted by anthropogenic activities, including nutrient enrichment from agriculture and urbanization, making it a critical region for investigating ecosystem responses to environmental change. While previous studies have explored virioplankton dynamics and their correlation with nutrient availability in the PRE [8, 11], comprehensive analyses of virus-host interactions and their functional roles in biogeochemical cycles remain scarce.

Understanding virus-host interactions is essential for unraveling mechanisms driving microbial ecology and nutrient cycling in estuarine ecosystems. Viruses influence microbial communities through host lysis, nutrient redistribution via the viral shunt, and the expression of auxiliary metabolic genes (AMGs) that modify host metabolism [5, 12]. Advances in metagenomics and machine learning have revolutionized the identification of novel viral taxa and the prediction of virus-host relationships, revealing intricate ecological networks in global oceans [13, 14]. However, research has largely focused on open oceans [15] or specific estuarine systems like the Chesapeake Bay [16] and Columbia River estuary [17]. These studies often examine individual microbial domains in isolation, overlooking the cross-domain interactions that are critical in estuarine ecosystems, where freshwater and marine environments converge to form unique ecological niches.

Recent advancements in sequencing technology now enable integrated analyses of microbial and viral communities through metagenomics [9, 16], uncovering previously unknown virus groups and their functional roles. Specifically, virus-encoded AMGs have been shown to influence host metabolic pathways, including nutrient cycling processes critical for ecosystem functioning [12, 18, 19]. The PRE, which supports a population of over 70 million people and spans diverse environmental gradients, represents a unique system to apply these cutting-edge approaches. While relationships between virioplankton abundance and nutrient dynamics in the PRE have been documented [8, 11, 20], fundamental knowledge gaps remain regarding virus-host interactions and their contributions to biogeochemical cycles [21, 22].

This study addresses these knowledge gaps by conducting a systematic evaluation of the entire microbial and viral community across the PRE. Using 24 metagenomic libraries from eight sampling stations, we assess how environmental gradients shape coupled dynamics of this complete assemblage, including viral and microbial community composition, host associations, and functional roles in biogeochemical cycles. By recovering high-quality viral genomes and predicting associated host interactions, we reveal key ecological patterns, including the dominance of Uroviricota, the unexpectedly high abundance of nucleocytoplasmic large DNA viruses (NCLDVs) in offshore waters, and the significant role of AMGs in modulating nitrogen and phosphorus cycling. This holistic, multidomain investigation highlights the importance of estuarine ecosystems as hotspots of viral diversity and provides critical insights into the complex interplay between environmental gradients, viruses, and their hosts.

## Materials and methods

### Sample collection

Eight sampling stations were established along a transect of the PRE (Table S1). All samplings were conducted in July 2021. At each station, duplicate 20 L water samples were collected from the Deep Chlorophyll Maximum (DCM) layer identified using real-time fluorescence profiles. Water samples were initially pre-filtered through a 50 μm nylon mesh (SEFAR, NITEX 03–50/31) to remove larger planktonic organisms. Sequential filtration was performed using a peristaltic pump system. The filtration cascade employed a 0.8 μm polyethersulfone membrane (142 mm diameter, Sterlitech), followed by a 0.2 μm polyethersulfone membrane (142 mm diameter, Sterlitech). Both membrane filters were immediately preserved in sterile 15 ml Falcon tubes, flash-frozen in liquid nitrogen, and stored at −80°C until DNA extraction.

The 0.2 μm filtrate underwent viral concentration using iron chloride flocculation following the established protocol [23]. Specifically, the filtrate was treated with iron chloride (final concentration 10 mg/L FeCl_3_), and the resulting viral precipitate was collected on a 142-mm, 1.0 μm polycarbonate membrane filter. The membrane was stored in a tinfoil-wrapped falcon tube at 4°C until further processing. Environmental parameters including temperature, salinity, conductivity, dissolved oxygen, and fluorescence were measured in situ using a CTD profiler. Additional water samples were collected for analysis of dissolved inorganic nutrients, flow cytometry measurements, and chlorophyll-a concentrations, which were analyzed as described previously [24, 25].

### DNA extraction, library construction, and sequencing

For cellular fractions (0.8 μm and 0.2 μm filters), membranes were cut into small pieces and immediately submerged in DNA/RNA Shield to preserve nucleic acid integrity [26]. DNA was extracted using the ZymoBIOMICS DNA/RNA Miniprep Kit, following the manufacturer’s protocol.

For viral fractions, membrane filters were incubated on a rotator overnight at 4°C in a freshly prepared resuspension buffer (0.1 M EDTA, 0.2 M MgCl_2_, 0.2 M ascorbate) with the an amount of 1 ml buffer for 1 mg Fe. To eliminate potential contamination from free DNA, the resuspended precipitate were treated with DNase I (100 U/ml) at room temperature for 2 hours, followed by enzyme inactivation using 0.1 M EDTA and 0.1 M EGTA [13]. Then the samples were concentrated using Amicon Ultra-15 centrifugal devices. Viral DNA was subsequently extracted using Wizard Columns and eluted in TE buffer.

The extracted genomic DNA was sent to Novogene (Tianjin, China) for library construction and sequencing. Genomic DNA quality was verified using 1% agarose gel electrophoresis. The genomic DNA was then fragmented to 300–500 bp using an M220 Focused-Ultrasonicator (Covaris Inc., USA). Paired-end libraries were constructed using NEXTFLEX Rapid DNA-Seq 2.0 (Bioo Scientific, USA), following the manufacturer’s protocol. Finally, the prepared libraries were sequenced using the Illumina Novaseq 6000 PE150 platform (Illumina Inc., USA).

### Metagenomic assembly, taxonomic classification, binning, and annotation

Raw metagenomic reads from 24 samples (16 cellular and 8 viral) (Table S2) were quality-filtered using Trimmomatic v0.39–2 [27] with parameters “TruSeq2-PE.fa:2:40:15 SLIDINGWINDOW:10:25 MINLEN:50”. Trimmed reads for each sample were individually assembled using metaSPAdes v3.13.1 [28] with default settings, generating in 24 assemblies (Table S3). Trimmed reads from cellular fractions were classified using Kaiju v1.9.2 [29] in mem-mode against the nr_euk database (May 2023), which encompasses bacteria, archaea, viruses, fungi, and microbial eukaryotes. Relative abundances at the phylum and class levels were computed using kaiju2table.

The OTU table is generated based on the read counts annotated to prokaryotic or eukaryotic species. To ensure the rigor of the analysis and avoid the overlap between viral operational taxonomic units (vOTUs) and OTUs, virus reads were removed before the prokaryotic and eukaryotic population analysis by employing bbmap v38.96 [30] and CoverM v0.4.0 [31] to map reads back to virus contigs with parameter “—min-read-percent-identity-pair 0.95—min-read-aligned-percent-pair 0.75”. This approach effectively separated high-confidence viral reads from cellular reads, creating a virus-depleted dataset for downstream prokaryotic and eukaryotic analyses while maintaining the integrity of cellular community composition data (Table S4).

As a supplement analysis for unclassified species which is not included in OTU table, Phyloflash v3.4.2 [32] was used to extract SSU rRNA (16S and 18S) reads and generate contigs from samples based on the SILVA database (release 138.1) [33]. The assembled SSU contigs from each sample and the reference sequence from the database with hits from our samples were clustered at 97% identity using CD-HIT v4.8.1 [34], generating 391 non-redundant OTUs. The extracted reads from phyloflash were then mapped back to the OTUs by using the “contig” mode in Coverm v0.4.0 [31] with the following parameters “contig—min-read-percent-identity 0.95—proper-pairs-only”. The taxonomy annotation of OTUs was done using SINA (v1.2.12) (https://www.arb-silva.de/aligner/) and Blastn.

Genome binning was performed on cellular metagenomes using MetaWrap v1.3.2 [35], integrating results from MaxBin2 v2.2.6 [36], MetaBAT v2.12.1 [37], and CONCOCT v1.0.0 [38]. Bins were refined using MetaWrap’s bin_refinement module and quality-assessed using CheckM v1.0.12 [39]. High-quality bins (≥50% completeness, ≤10% contamination) were retained, excluding non-planktonic Rickettsiales bins.

The initial set of 1164 MAGs (1076 bacterial, 88 archaeal) was dereplicated using Drep v3.4.2 [40] with default parameters (95% average nucleotide identity [ANI], ≥75% completeness, ≤10% contamination), yielding 104 bacterial and 13 archaeal representative MAGs. For the classification of these MAGs, the GTDB-tk v2.4.1 [41] was used, applying metrics such as relative evolutionary divergence and ANI to ensure precise taxonomic placement. MAG abundances were estimated using CoverM v0.4.0 [31] with parameters “—min-read-aligned-length 50—min-read-percent-identity 0.95—min-covered-fraction 0—proper-pairs-only”. Open reading frames were predicted using Prodigal v2.6.3 [42] and annotated using DRAM v1.3.4 [43] against the KEGG, Pfam, CAZy, and VOGDB databases to characterize metabolic potential.

### Viral genome recovery and analysis

Following the Sullivan Lab protocol [44], viral contigs were identified from assembled contigs using VirSorter2 v2.2.3 [45] with parameters “—include-groups dsDNAphage, NCLDV, ssDNA—min-length 5000—min-score 0.5—keep-original-seq”. To ensure the recovery of high-confidence viral genomes suitable for downstream analysis, we adopted a conservative identification strategy centered on VirSorter2. Its precision-oriented, multi-classifier framework is effective at minimizing the inclusion of non-viral mobile genetic elements common in complex estuarine metagenomes, thereby strengthening the validity of subsequent predictions. Initial predictions were refined using CheckV v0.8.1 [46] to remove host genes and filter proviruses, followed by a second VirSorter2 pass with parameters “-seqname-suffix-off—viral-gene-enrich-off—provirus-off—prep-for-dramv” to prepare for DRAM-v annotation. DRAM-v v1.3.4 [43] was used to annotate the identified viral sequences. Sequences were retained if they contained viral genes or met quality thresholds (alignment score ≥ 0.95, >2 hallmark viral features, or absence of host genes). Sequences >10 kb containing host genes but lacking viral genes underwent manual curation.

The identified 50 019 viral contigs were clustered into species-level vOTUs according to MIUViG guidelines using cd-hit v4.8.1 at 95% identity and 85% coverage [34, 47, 48]. This yielded 29 952 clusters, with the longest contig selected as the representative for each cluster. CheckV assessed contig quality, with ≥90% completeness classified as high-quality. VIBRANT v1.2.1 [49] and bacphlip v0.9.6 [50] predicted viral lifestyle (temperate/virulent).

Open reading frames (ORFs) within the viral contigs were predicted by Prodigal v2.6.3 [42]. Taxonomic classification was performed using CAT v5.2.3 [51] against Viral RefSeq214 with default parameter (r = 10, f = 0.50). We chose CAT for its protein-homology-based algorithm, which provides more robust classification for the novel and divergent viral sequences common in environmental metagenomes compared to k-mer-based methods. To ensure high-confidence assignments, we implemented a stringent two-step process. First, an initial classification was generated by CAT. Second, we required this assignment to be supported by annotations on >50% of the ORFs on a given contig. If a contig passed the first step but failed this ORF-support threshold, it was conservatively downgraded to a “potential” higher-level taxon (e.g. potential *Uroviricota*). Contigs that could not be classified by CAT in the first step remained designated as “unclassified”. This rigorous approach prevents over-classification of novel sequences based on limited evidence. Viral abundance was determined by mapping reads using bbmap v38.96 [30] and filtering with CoverM v0.4.0 [31] at >95% identity and >75% alignment, calculating FPKM for contigs with >70% coverage.

For virus-prokaryotic host predictions, iPHoP v1.3.3 [52] analyzed 714 high-quality viruses (>90% completeness) against a comprehensive host database (iPHoP_db_Aug23_rw, incorporating GTDB r214, IMG genomes as of Aug 2023, MGnify MAGs, GEMv1, and 117 recovered MAGs). Host predictions were retained with confidence scores ≥90 and visualized using Cytoscape v3.9.1. Then, DRAM-v v1.3.4 [43] was used to predict AMGs, filtering with an auxiliary score ≤ 3 and excluding specific amg_flag categories (T, V, A, P, B). To account for potential host DNA contamination, viral-encoded AMG abundances were estimated using vOTU abundances as proxies.

### Statistics analysis and data visualization

Statistical analyses were performed using R package vegan [53]. For alpha diversity assessment, Shannon and Inverse Simpson indices were calculated from flattened read counts for prokaryotic and eukaryotic communities, with Pearson correlation employed to identify environmental drivers of biodiversity. Beta diversity analysis utilized PCoA based on Bray–Curtis dissimilarity matrices, while PERMANOVA was used to test group significance between estuarine and oceanic communities. Similar analyses were conducted for viral communities using FPKM data. The relationships between environmental factors and community structure were examined using Mantel’s test and Canonical Correspondence Analysis (CCA). To identify distinct metacommunities, Dirichlet multinomial mixtures (DMM) analysis was performed using flattened read counts as input. The optimal number of clusters (*n* = 2) was determined by comprehensively considering the Laplace approximation score, Akaike Information Criterion and Bayesian Information Criterion. Samples were subsequently assigned to two distinct envirotype, with taxon contribution values calculated based on their relative abundance. Results visualization was accomplished using the online platform ChiPlot [54] and R packages, including ggplot2 [55], ggpubr [56], and ggsci [57].

## Results and discussion

### Distinct responses of microbial and viral communities to environmental gradients

**Figure 1:**
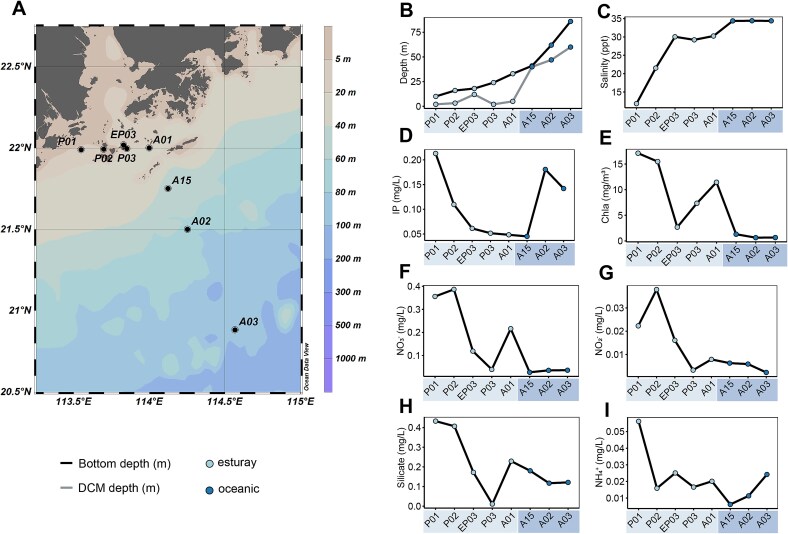
Geographical distribution and environmental gradients of the Pearl River estuary. The map in (A) shows the locations of five estuarine sampling sites near the river mouth (P01, P02, EP03, P03, A01) and three oceanic sites (A15, A02, A03) extending into the South China Sea. Bathymetric contours illustrate depth variations, ranging from the shallow estuary to deeper oceanic waters. Panels B–I present the environmental parameter profiles along the sampling transect: (B) water depth, showing the bottom depth and the position of the deep chlorophyll maximum (DCM); (C) salinity gradient; (D) IP concentrations; (E) chlorophyll *a* concentrations; (F) nitrate (NO₃^−^) concentrations; (G) nitrite (NO₂^−^) concentrations; (H) silicate concentrations; and (I) ammonium (NH₄^+^) concentrations.

The PRE exhibited strong physicochemical gradients during the summer of 2021, creating distinct environmental zones (Fig. 1, Table S1). Salinity increased seaward from 11.8‰ to 34.4‰, while nutrient concentrations showed opposing trends, with nitrate decreasing from >0.38 mg/L in the inner estuary to <0.03 mg/L at the outer stations. Phosphate followed a similar spatial pattern. Surface temperatures ranged from 22.1–30.3°C, with thermal stratification of up to 7.6°C observed between surface waters and the DCM layer at the outer stations (Table S4). Hierarchical clustering of these parameters delineated two distinct zones: an estuarine zone (stations P01-P03, EP03) characterized by lower salinity (<31‰) and higher nutrient concentrations, and an oceanic zone (stations A01-A03, A15) with contrasting characteristics (Table S5). This zonation aligns with previous studies identifying the PRE as a major nutrient source to the northern South China Sea [1], providing a framework for examining microbial community distributions.

**Figure 2:**
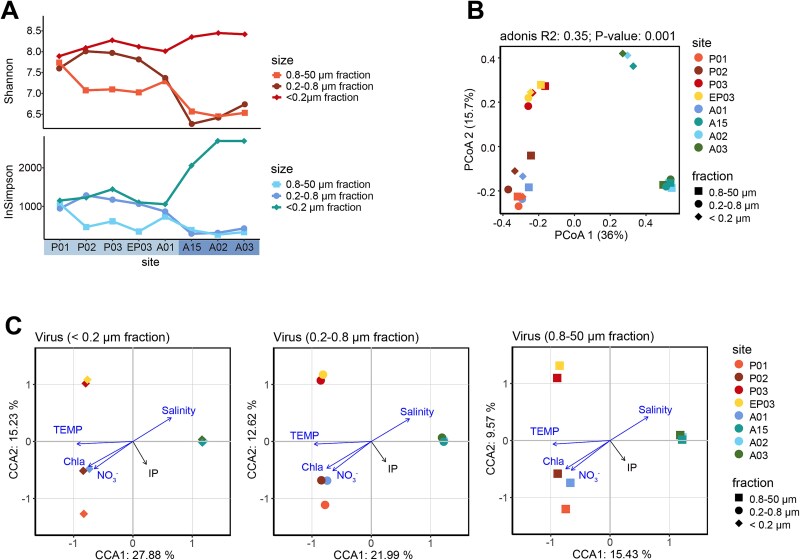
Viral community diversity patterns and environmental drivers across the Pearl River estuary. (A) Alpha diversity indices showing Shannon (top) and inverse Simpson (bottom) indices for viral communities across the eight sampling sites in three size fractions (0.8–50 μm, 0.2–0.8 μm, <0.2 μm) based on normalized read counts. (B) PCoA analysis of viral community compositions demonstrating significant clustering by water body type, distinguishing estuarine and oceanic waters (Adonis R^2^ = 0.42, *p* < 0.05). (C) CCA illustrates relationships between viral community structures and measured environmental factors, including temperature, chlorophyll *a*, salinity, IP, and nitrate (NO₃^−^). Vectors indicate significant environmental drivers (*p* < 0.05) as determined through marginal effects testing.

Microbial diversity patterns along the estuary-ocean gradient mirrored these environmental zones. Both Shannon and Inverse Simpson diversity indices indicated consistently higher microbial diversity in nutrient-rich estuarine waters compared to oceanic waters (Figs S1A, S2A). PCoA further demonstrated significant separation between estuarine and oceanic zones for both prokaryotes (Adonis *R^2^* = 0.42, *p* = 0.001) and eukaryotes (Adonis *R^2^* = 0.4, *p* = 0.001) (Figs. S1B, S2B). Viral communities, however, showed more nuanced distribution patterns, varying by size fraction (Fig. 2). While cellular fractions (0.2–0.8 μm, 0.8-50 μm) displayed reduced diversity offshore, free-living viral fractions (<0.2 μm) exhibited higher diversity in oceanic waters (Fig. 2A). PCoA (adonis *R^2^* = 0.35, *p* = 0.001) identified four distinct clusters corresponding to different environmental zones and size fractions: offshore free-living viruses, offshore cellular fractions, main estuarine group, and a separate estuarine cluster (Fig. 2B).

**Figure 3:**
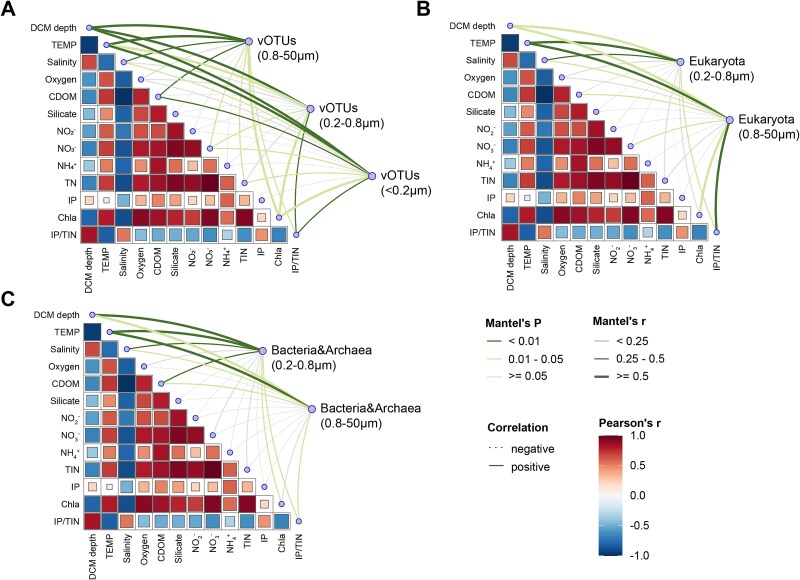
Relationships between environmental factors and virus/host communities. (A) Correlations between environmental factors and viral communities across three size fractions (<0.2 μm, 0.2–0.8 μm, and 0.8–50 μm). (B) Correlations between environmental factors and eukaryotic communities. (C) Correlations between environmental factors and bacterial and archaeal communities. Heatmaps display Pearson’s correlation coefficients among environmental variables, while lines represent mantel test (based on Pearson correlation) results. Line thickness indicates correlation strength (Mantel’s *r*), and color denotes statistical significance: a high level of significance (p < 0.01), moderate significance (0.01 ≤ p < 0.05), and no significant correlation (p ≥ 0.05). Environmental parameters include temperature (TEMP), salinity, dissolved oxygen, colored dissolved organic matter (CDOM), nutrients (silicate, NO₃^−^, NO₂^−^, NH₄^+^, TIN, IP), chlorophyll *a* (Chla), and the IP/TIN ratio.

To investigate the drivers of these community patterns, we conducted correlation analyses between community composition and measured physicochemical parameters. Based on the results of the normality tests (Fig. S3), not all variables statistically conform to a normal distribution. Therefore, we applied both Pearson and Spearman correlation coefficients to make the results more robust. Mantel’s test revealed that environmental parameters significantly influenced the distribution of all microbial groups: prokaryotes, eukaryotes, and viruses, demonstrating consistent impacts across domains (r > |0.25|, *p* < 0.05) (Fig. 3, Fig. S4). Key environmental drivers included temperature, salinity, CDOM, nitrogen sources, chlorophyll-a and TIN/IP. Pearson and Spearman correlation analysis further highlighted domain-specific responses: prokaryotic diversity correlated with salinity, indicating salinity as a key selective force, whereas eukaryotic diversity was primarily influenced by water temperature (Figs S5, S6).

CCA based on read counts provided further insights into the environmental gradients shaping microbial communities. Focusing on four key variables, nitrate, chlorophyll-α, temperature and inorganic phosphorus (IP). CCA revealed clear gradients driving community separation. Salinity increased, while temperature, chlorophyll-a, and nitrate declined toward oceanic waters (Figs 2C, S1C, S2C). Although IP demonstrated minimal direct impact in earlier analyses, its inclusion in CCA was justified by the PRE’s known phosphorus limitation and potential indirect effects through host taxa. The dominant role of salinity in structuring prokaryotic communities aligns with observations from other major estuarine systems [58, 59]. In contrast, viral communities exhibited stronger responses to nutrient availability, with higher nutrient levels correlating with reduced viral diversity [13, 14]. These results emphasize how environmental gradients differentially shape microbial and viral communities, with each group displaying distinct responses to the estuarine-oceanic transition. It is important to acknowledge that our analysis of bacterial and eukaryotic microbial communities was conducted using taxonomically annotated reads (Tables S6, S7), which inherently excludes non-annotatable species due to incomplete reference database coverage. However, compared to traditional amplicon-based approaches targeting 16S and 18S rRNA genes (Table S8), our shotgun metagenomic methodology captures a substantially broader taxonomic range by utilizing all sequencing reads rather than being restricted to specific phylogenetic markers. Leveraging read-based methods, 73.3% of the prokaryotic reads and 84.35% of the eukaryotic reads on average were assigned to species and involved in the following statistical analysis (Table S9). This approach avoids primer bias and low marker gene copy number limitations inherent in amplicon sequencing, enabling detection of species potentially overlooked by traditional methods. While we acknowledge the annotation bias toward well-characterized organisms in reference databases, our methodology provides a more comprehensive understanding of microbial diversity patterns than achievable through marker gene approaches, particularly for capturing the full breadth of microbial life in complex estuarine-marine environments.

### Partitioning of prokaryotic and eukaryotic ecotypes along the PRE gradient

The environmental gradients observed in PRE were closely reflected in the distribution and composition of microbial communities. Taxonomic composition, based on relative read abundance, revealed that bacteria dominated the prokaryotic communities in both size fractions (54.61% in 0.2–0.8 μm; 29.68% in 0.8–50 μm) (Fig. S7). Among bacterial taxa, Pseudomonadota (49.19%) and Bacteroidota (21.36%) were the predominant phyla (Fig. S8B). Flow cytometry further confirmed bacteria were the dominant group in terms of absolute abundance (Fig. S9C). Moving along the salinity gradient toward offshore waters, we observed an increasing proportion of archaea and eukaryotes, with the archaeal community dominated by Thermoplasmatota (40.79%), Methanobacteriota (23.46%) and Nitrososphaerota (20.21%) (Figs. S4, S5). This shift in community composition, with a higher archaeal abundance in more saline waters, is consistent with observations from other estuarine systems [17, 60].

DMM analysis of read-based data classified the eight sampling sites into two distinct envirotypes for prokaryotic communities: estuarine (stations P01, P02, P03, EP03, A01) and oceanic (stations A02, A15, A03), consistent with the PCoA analysis results (Fig. S1). The estuarine ecootype is predominantly composed of bacterial species, with the top 14 of 15 contributors being prokaryotic bacteria. In contrast, the oceanic envirotype shows significant contributions from both bacteria and archaea (Fig. S10). *Flavobacteriales bacteriu*m, *Alphaproteobacteria bacterium, Gammaproteobacteria bacterium, Actinomycetota bacterium, Candidatus Poseidoniales archaeon* were identified as highly abundant across both envirotypes, underscoring its remarkable ecological adaptability (Fig. S10, Tables S10, S11).

**Figure 4:**
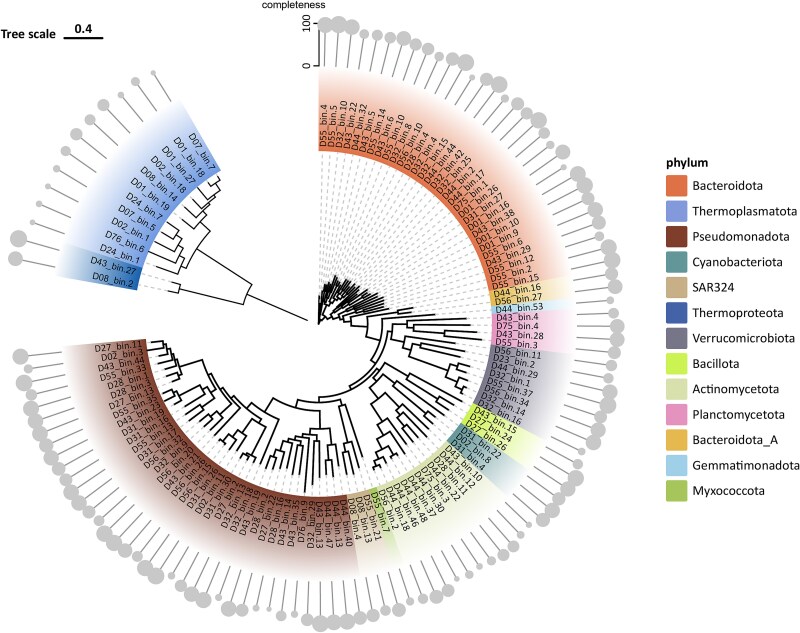
Phylogenetic analysis of recovered bacterial and archaeal metagenome-assembled genomes (MAGs) from the Pearl River estuary. (A) the circular phylogenetic tree illustrates the evolutionary relationships among the recovered MAGs. (B) Archaeal MAGs are positioned in the upper left quadrant (blue shading), while bacterial MAGs occupy the remainder of the tree. Major bacterial phyla are color-coded according to the legend, providing clear taxonomic differentiation. (C) the outer circles represent genome completeness, measured on a scale from 0 to 100%.

**Figure 5:**
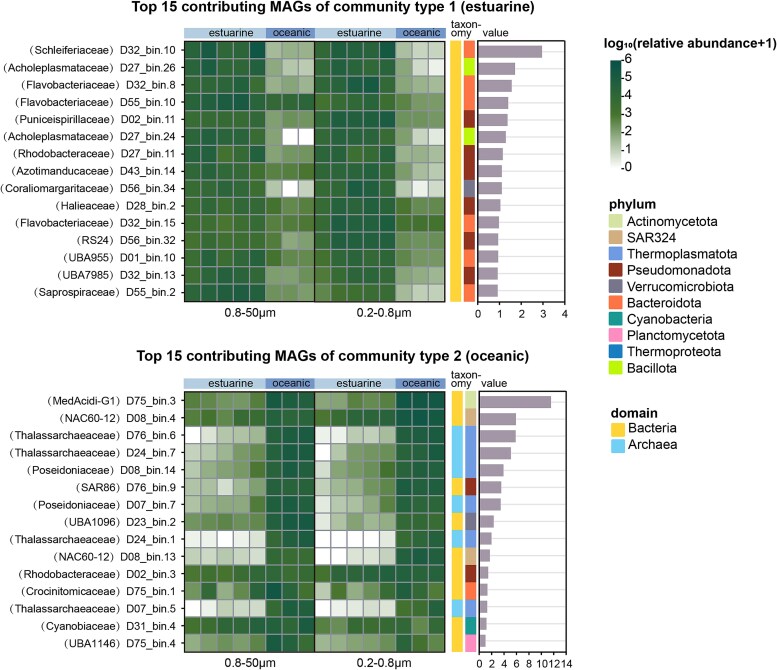
DMM analysis of bacterial and archaeal MAGs across the Pearl River estuary. (A) DMM clustering identified two distinct prokaryotic ecotypes: Estuarine (top panel) and oceanic (bottom panel), highlighting the top 15 contributing MAGs for each community type. (B) A heatmap displays the relative abundance of these MAGs across the eight sampling stations in two size fractions (0.8–50 μm and 0.2–0.8 μm). Taxonomic classifications are provided with family names in parentheses, and color bars indicate phylum and domain affiliations. (C) The rightmost bar charts illustrate the contribution values of each MAG to its respective community type, emphasizing the ecological roles of these taxa.

The metagenomic assembly yielded 117 high-quality MAGs, comprising 104 bacterial and 13 archaeal MAGs, mirroring the patterns observed in the read-based analyses (Fig. 4). The predominant phyla were Pseudomonadota (37 MAGs) and Bacteroidota (31 MAGs), collectively accounting for 60% of all recovered MAGs (Fig. 4). Additionally, DMM analysis of these MAGs reaffirmed the division into estuarine and oceanic ecotypes, further validating the observed ecological patterns (Fig. 5, Table S10). By combining the classification results from read-based and MAG-based approaches, we not only compensate for the shortcomings of read-based classification but also include a large number of reads that cannot be assembled into MAGs, making the results more comprehensive.

Consistent patterns emerged across both approaches for major bacterial groups (Fig. S10, Table S11). Within Alphaproteobacteria, *Rhodobacteraceae bacterium HIMB11, Rhodospirillaceae bacterium, JADHLI01 sp038121125* (D02_bin.11)*, LFER01 sp001510135* (D27_bin.11)*, and UBA8337 sp009920875* (D56_bin.32) were dominant in estuarine environments, whereas *Paracoccaceae bacterium and Salinivivens sp001627375* (D02_bin.3) prevailed in oceanic waters. A similar habitat-specific distribution was evident in Gammaproteobacteria, with *SAR86 cluster bacterium* and *GCA-2707915 sp902570225* (D76_bin.9) characterizing oceanic communities, while *Halieaceae bacterium*, *SAR86 cluster bacterium SAR86B* and dominated estuarine waters. In Cyanobacteria, *Synechococcus* thrived in estuarine regions, whereas *Prochlorococcus* was predominant in oceanic waters, consistent with their well-documented global distributions [61].

The archaeal community showed remarkable consistency between read-based and MAG analyses. Both approaches demonstrated an increase in archaeal diversity and abundance toward oceanic waters, with Thermoplasmatota*, which,* Methanobacteriota as the predominant phyla in oceanic water (Fig. S8C). This trend was particularly evident in the MAG dataset, where *Thermoplasmatota* accounted for 9 of the 13 archaeal MAGs recovered (Fig. 4, Table S10).

The eukaryotic community structure, characterized primarily through read-based analyses due to limitations in MAG recovery, showed distinct spatial organization. In offshore waters, the 0.2–0.8 μm size fraction was dominated by Chlorophyta (>90%), particularly *Ostreococcus* species, while the larger size fraction (0.8–50 μm) showed co-dominance of Chlorophyta (48.6%–57.06%) and Pelagophyceae (28.05%–33.78%) (Figs S8A, S11, Table S12). In contrast, the estuarine envirotype was dominated by diatoms, including *Chaetoceros tenuissimus* and *Thalassiosira* species (Fig. S11, Table S12). The inability to recover high-quality eukaryotic MAGs, likely due to the larger genomes and greater complexity of eukaryotic organisms, underscores the continuing challenges in achieving comprehensive genomic characterization of marine microbial communities.

### Viral genome recovery and classification

**Figure 6:**
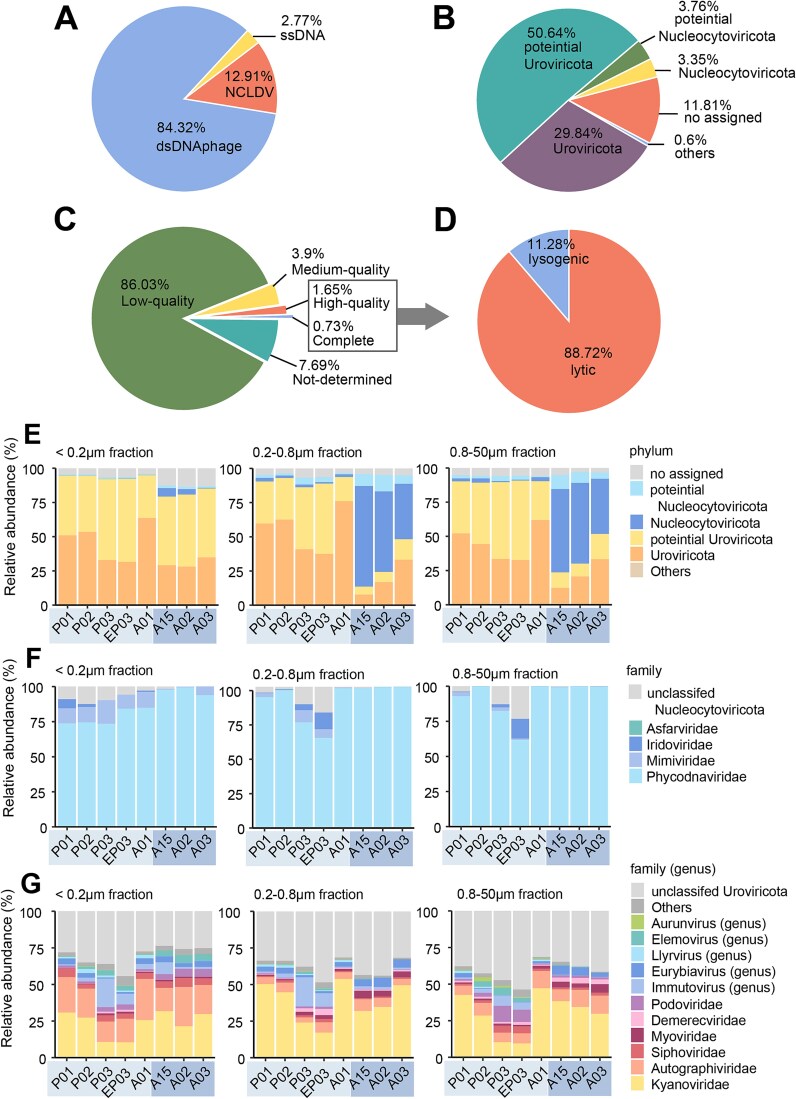
Taxonomic composition, genome quality, and ecological strategies of viruses in the Pearl River estuary. (A) Relative abundance of major virus types. (B) Relative abundance of the two major viral phyla identified in the population, *Uroviricota* and *Nucleocytoviricota*. (C) Quality assessment of viral genome assemblies, categorized as low-quality (<50%), medium-quality (50–90%), high-quality (90–100%), and complete (100%) genomes. (D) Life strategy distribution (lytic vs. lysogenic) among high-quality and complete viral genomes, with viruses lacking evidence of lysogeny assumed to be lytic. (E) Phylum-level viral community composition across three size fractions (<0.2 μm, 0.2–0.8 μm, 0.8–50 μm) at each sampling site. (F) Family-level distribution within *Nucleocytoviricota* across size fractions and sampling sites. (G) Family/genus-level distribution within *Uroviricota* across size fractions and sampling sites.

To complement our understanding of cellular microbial communities, we next examined the viral component of these ecosystems in the PRE. From 24 metagenomic libraries spanning eight sampling sites and 3 size fractions (0.8-50 μm, 0.2–0.8 μm, <0.2 μm), we recovered 59 019 non-redundant viral contigs (>5000 bp). These contigs were clustered into 29 952 vOTUs at 95% similarity. Double-stranded DNA bacteriophages dominated the viral community (84.2%), followed by Nucleocytoplasmic Large DNA Viruses (NCLDVs; 12.91%) and single-stranded DNA viruses (2.77%) (Fig. 6A). Quality assessment using CheckV revealed that 86.03% of contigs were low quality (<50% completeness), while only 1.65% were classified as high quality (≥90% completeness), and 0.73% represented complete genomes (Fig. 6C). This quality distribution aligns with findings from previous marine virome studies, which report similarly low completion rates for seawater viromes [62] and deep-sea sediments [63]. These low completion rates reflect the challenges in assembling viral genomes from environmental samples, primarily which are compounded by high viral diversity, rapid mutation rates, and technological limitations. Such fragmented assemblies constrain our ability to fully characterize viral functions and host interactions in marine ecosystems. Nevertheless, these high-confidence genomes formed the foundation for our subsequent in-depth analyses, including lifestyle prediction, host interaction networks, and metabolic profiling.

Detailed analysis of the viral contigs revealed a distinct dominance of tailed bacteriophages, with 29.84% classified as *Uroviricota* and 50.64% as potential *Uroviricota*. Giant viruses, represented by NCLDVs, accounted for a smaller but significant fraction (3.35% *Nucleocytoviricota*, 3.76% potential *Nucleocytoviricota*), while 11.88% of the contigs remained unclassified (Fig. 6B). This classification pattern aligns with previous marine virome studies [14], though methodological biases may underrepresent non-tailed viruses [64]. The high proportion of potential classifications highlights the substantial unexplored viral diversity present in marine environments. From our dataset, we identified 714 high-quality viral genomes (≥90% completeness), with genome lengths predominantly around 50 000 bp. Among these, 88.72% were predicted to exhibit a lytic lifestyle, while 11.28% were lysogenic (Fig. 6D). These ratios are consistent with environmental bacteriophage distributions reported by Luo *et al.* [10] and reflect fundamental ecological strategies of viral communities. However, it is important to note that these results may not fully represent single-stranded DNA (ssDNA) viruses, as their detection and classification remain limited by current methodological approaches.

### *Uroviricota* dominance across free and potential host-associated viral communities

Further examination of *Uroviricota* revealed their dominance across all sampling sites, with the highest abundance in the free virus fraction (<0.2 μm; 79.07%–94.83%).

and substantial presence in cellular fractions (0.2–0.8 μm, 0.8–50 μm; 85.91%–93.32%) of estuarine environments (Fig. 6E). Within this phylum, we identified 16 prominent families or genera, each constituting >1% of the viral community in at least one sample, based on Refseq214 and NCBI taxonomy browse (October 2022). A significant portion of *Uroviricota* (36.59% average) remained unclassified, highlighting the vast diversity of undiscovered marine viruses (Fig. 6G).

Among the classified taxa, *Kyanoviridae* and *Autographiviridae* were the most dominant families, followed by *Immutovirus*, *Eurybiavirus*, and the traditional tailed-phage families (*Siphoviridae*, *Podoviridae*, and *Myoviridae*) (Fig. 6G). *Kyanoviridae*, a recently established taxon representing T4-like cyanophages, dominated cellular fractions (30.60% on average), likely reflecting their broad host range and significant ecological impact. In contrast, *Autographiviridae* (T7-like phages) were primarily found in the free virus fraction (11.78% on average), suggesting an adaptation to persist in the water column despite their relatively narrow host range. The presence of *Uroviricota* in both free-living and host-associated states indicates diverse life strategies, including active infection cycles and potential lysogenic integration into host genomes. This ability to switch between lytic and lysogenic cycles enables these viruses to exert significant influence on host population dynamics, metabolic processes, and nutrient cycling, thereby contributing to microbial community stability and resilience.

### Unexpectedly high NCLDV abundance in offshore waters

In contrast to the global dominance of bacteriophages (*Uroviricota*) in marine environments [9, 65], our offshore samples revealed an unusually high abundance (44.86–46.63%) of *Nucleocytoviricota*, a group of eukaryote-infecting viruses also known as nucleocytoplasmic large DNA viruses (NCLDVs), in both cellular fractions (0.2–0.8 μm and 0.8–50 μm) (Fig. 6E). This far exceeds the typical ~3% NCLDV abundance reported in Tara Ocean surveys [66]. However, such dramatic fluctuations are consistent with previous observations of specific NCLDVs, such as *Ostreococcus tauri* viruses, which can range from undetectable to high abundance depending on environmental conditions [67]. These variations were particularly pronounced in our offshore stations (A15, A02, and A03), reflecting the dynamic nature of marine viral populations.

The elevated NCLDV abundance correlated with higher levels of their eukaryotic hosts, with eukaryotes constituting 3.41% (0.2–0.8 μm) and 5.00% (0.8–50 μm) of the total microbial community (Fig. S7). Chlorophyta dominated these eukaryotic populations, representing 52.38% and 94.15% in the respective size fractions (Fig. S8). This substantial presence of green algae, which are known hosts for many NCLDVs, explains the distribution pattern observed. While nearshore stations were predominantly populated by bacteria, offshore sites harbored more eukaryotic hosts suitable for NCLDV infection. This spatial distribution of host communities explains the unusually high NCLDV counts in the offshore samples, highlighting the critical role of host availability in viral ecology.

Detailed taxonomic analysis revealed distinct viral compositions across sampling sites, including several abundant genera that did not align with known viral families. *Phycodnaviridae* dominated the NCLDVs, comprising 89.34% across all locations. Other NCLDV families showed habitat-specific distribution: *Iridoviridae* were primarily confined to estuarine waters’ free-living viral fraction (<0.2 μm) and both small (0.2–0.8 μm) and larger (0.8–50 μm) fractions in transitional waters, whereas *Mimiviridae* exhibited broader distribution across all size fractions (Fig. 6F). These distributions partially align with global surveys reporting significant contributions of large viruses (25.4% in <0.2 μm, 29.7% in 0.22–3.0 μm [68]). However, our offshore samples showed notably higher *Phycodnaviridae* representation and lower *Mimiviridae* abundance, likely reflecting specific environmental conditions in offshore areas that support the unusual prevalence of large viruses.

To better understand these distribution patterns, we analyzed viral sequences from eight sampling sites using RPKM quantification and Shannon diversity indices (Fig. 2A). The analysis revealed distinct size-fraction patterns: cellular fractions (0.2–0.8 μm, 0.8–50 μm) showed reduced viral diversity in offshore waters, but free-living viral fractions (<0.2 μm) exhibited higher diversity compared to estuarine waters. PCoA demonstrated significant differences in community composition across water types (adonis *R^2^* = 0.35, *p* = 0.001; Fig. 2B). Four distinct clusters were identified: offshore free-living viruses (<0.2 μm; stations A15, A02, A03), offshore cellular fractions (0.2–50 μm), main estuarine group (A01, P01, P02), and a separate estuarine cluster (P03, EP03). The separation between viral and cellular fractions in offshore waters reflects elevated NCLDV abundance in cellular fractions.

### Predicting virus-host relationships through genomic and machine learning based methods

**Figure 7:**
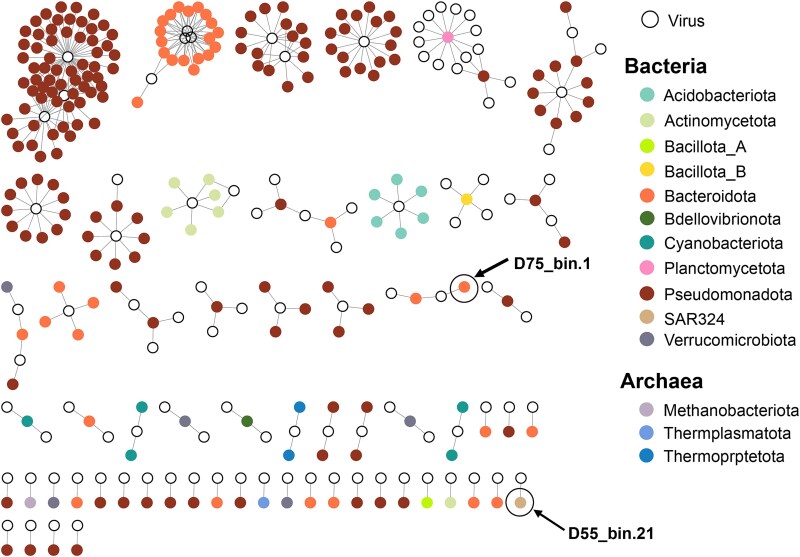
Virus-host pairs network. (A) Network visualization of putative virus-host associations predicted by iPHoP. Viruses are represented as white circles with black borders, connected to their predicted prokaryotic hosts, which are displayed as colored circles. (B) Colors indicate bacterial and archaeal phyla, as shown in the legend. Two novel potential hosts identified from the recovered MAGs (*D75_bin.1* and *D55_bin.21*) are specifically highlighted, showcasing key virus-host interactions in the PRE.

To elucidate how these environmental drivers influence specific biological interactions, we implemented a stringent prediction framework for virus-prokaryote relationships. Our approach focused on viral genomes with >90% completeness and integrated both phage-based and host-based machine learning based methods, utilizing a comprehensive host database combining public repository data with locally sequenced bacterial and archaeal genomes. This analysis revealed 348 virus-prokaryote connections, comprising 227 prokaryotic genomes/bins and 107 viral genomes, which we reorganized into 61 distinct clusters (Fig. 7, Table S13).

The viral component predominantly consisted of the *Uroviricota* phylum, with only three genomes remaining unclassified. Host diversity spanned 14 phyla, including three archaeal phyla (*Thermoplasmatota*, *Bathyarchaeia*, *Methanobacteria*) and 11 bacterial phyla (*Acidobacteriota*, *Actinomycetota*, *Bacillota_A*, *Bacillota_B*, *Bacteroidota*, *Bdellovibrionota*, *Cyanobacteriota*, *Planctomycetota*, *Pseudomonadota*, *SAR324*, *Thermoproteota*, *Verrucomicrobiota*). Among these, *Pseudomonadota* (*n* = 158) and *Bacteroidota* (*n* = 36) dominated the host predictions, collectively accounting for 85.8% of all connections. This dominance is consistent with their prevalence in our bacterial community profiles.

Two notable local virus-host interactions emerged from our dataset. The first involved an unclassified *Caudoviricetes* virus associated with *D55_bin.21* (SAR324 phylum, *JCVI-SCAAA005 sp011522845*species). SAR324 bacteria, known for their chemolithoautotrophic metabolism, play crucial roles in sulfur cycling and organic matter degradation in oxygen-minimal zones [69, 70]. The second interaction was observed in a cluster containing two viruses and two bacteria, where the local bacterial bin *D75_bin.1* shared bacteriophages with *GB_GCA_002696025*. Both strains belong to the *UBA952* genus (*Crocinitomicaceae* family), typically associated with organic matter processing in carbon-rich marine environments [71].

Network analysis revealed sophisticated interaction patterns, with 31 clusters containing multiple viral or prokaryotic genomes. These interactions exhibited nested and modular structures, where individual viruses infected multiple hosts, and single hosts were associated with multiple viruses [72]. The observed enrichment of *Uroviricota*-*Pseudomonadota* and *Uroviricota*-*Bacteroidota* pairs aligns with interaction patterns previously documented in marine biofilms and soils [73, 74]. While most virus-host infections occurred within individual phyla, we identified two instances of potential cross-phylum infections. Although cross-species and cross-genera viral infections are well-documented [75, 76], cross-phylum infections remain unverified under laboratory conditions. This discrepancy between computational predictions and experimental validation suggests the possibility of novel biological phenomena or methodological artifacts, necessitating further investigation through controlled experiments.

### Viral AMGs and host metabolism shape nutrient cycling

Building on the ecotype distributions and virus-host interactions described earlier, we investigated the metabolic capabilities of these systems to understand how they support the observed community patterns. Our analysis revealed that metabolic capabilities were closely aligned with the environmental zonation and community structure, with clear distinctions between estuarine and oceanic populations.

**Figure 8:**
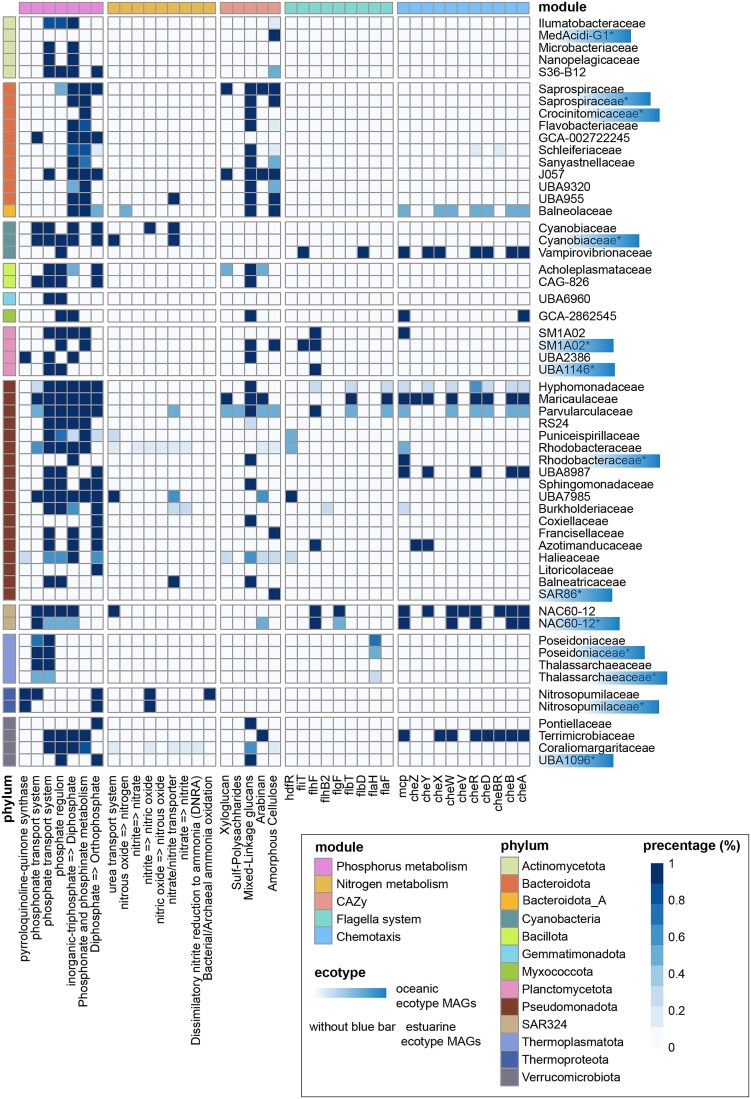
Functional gene distribution across bacterial and archaeal MAGs. The heatmap illustrates the proportion of MAGs within each taxonomic family (rows) containing key functional genes (columns) involved in biogeochemical cycling processes and environmental adaptation. Functional categories include nitrogen cycling, phosphorus cycling, organic carbon degradation, and motility/chemotaxis. Specific functional genes and pathways are listed in Table S15. Colored modules at the top indicate the functional groupings. Families belonging to the oceanic ecotype, as determined by DMM analysis, are highlighted on the right, while unmarked families represent the estuarine ecotype. Phylum-level taxonomy is indicated by the color bar on the left. Gene presence was determined using KEGG/CAZy database annotations, and the functional genes or pathways encoded by each MAG are detailed in Table S16.

Host metabolic analysis identified two fundamental functional categories reflecting adaptations to the environmental gradients in the PRE. The first category involved survival adaptation mechanisms, which were particularly enhanced in estuarine populations. Taxa such as *Balneolaceae*, *Hyphomonoadaceae*, and *Maricaulaceae* exhibited well-developed chemotaxis and flagellar systems (Fig. 8). These adaptations align with the dynamic conditions in estuarine waters, where rapid responses to fluctuating nutrients and salinity are critical [77, 78]**.** This finding corresponds to the higher prokaryotic diversity observed in nutrient-rich estuarine waters and highlights the ecological success of these organisms in environments with steep physicochemical gradients.

The second category involved biogeochemical cycling capabilities, which showed ecological partitioning consistent with the observed community composition. Estuarine ecotypes, dominated by bacterial species, demonstrated superior phosphorus cycling capabilities, averaging 3.4 out of 7 studied pathways compared to 1.7 in oceanic ecotypes (Mann–Whitney U test; *p* < 0.01) (Fig. S12). This enhanced metabolic capability aligns with the higher nutrient availability and metabolic demands in estuarine waters. Similarly, nitrogen cycling genes showed specialization patterns that mirrored the community structure. For example, *Nitrosopumilaceae*, which were more abundant in saline offshore waters, contained both ammonia monooxygenase (*amoABC*) and denitrification (*nirK*) genes, reflecting adaptations to the nitrogen-limited conditions characteristic of these regions (Fig. 8).

The viral component of these metabolic systems showed a high degree of alignment with host capabilities and environmental conditions. Among the 11 023 AMGs identified, spanning 160 metabolism-related pathways, our analysis revealed patterns suggesting a potential viral adaptation to the same environmental pressures that shape host communities (Fig. S13, Table S14). Phosphorus-related AMGs, including *phoH* (178 copies) and *pstS* (42 copies), appeared to mirror the enhanced phosphorus cycling capabilities of estuarine host communities. This points to a potential coordinated adaptation of hosts and viruses to phosphorus availability, a key environmental driver identified in our earlier analyses. The *phoH* gene likely encodes an ATPase that supports survival under low-phosphate conditions [79]**,** while *pstS* has been experimentally confirmed to enhance phosphate uptake [80], though its functional role in our system requires further validation.

Similarly, nitrogen metabolism AMGs (31 total, including 10 copies of *pomC-amoC* and 21 copies of *glnA*) reflected the prokaryotic ecotype boundaries identified earlier. The presence of *amoC* genes in viruses infecting ammonia-oxidizing hosts such as *Nitrosopumilus* (MAG *D43_bin.27*) suggests a potential role for viruses in supporting host nitrification capabilities, particularly important in nitrogen-limited offshore waters. However, as these conclusions are based on genomic potential, transcriptional analyses, such as qPCR, would be required to confirm these putative functional roles [19]**.**

In addition, we observed elevated levels of xenobiotics biodegradation-related AMGs compared to other marine environments [73]**.** This finding may reflect the anthropogenic influence on the PRE, as captured in our environmental characterization. The presence of these AMGs suggests that both hosts and viruses may have adapted to mitigate the unique pressures of this human-impacted ecosystem.

The integration of host metabolic functions and viral AMGs highlights sophisticated adaptations that could underpin the observed community structures and ecological patterns in the PRE. The alignment between host metabolic capabilities and viral AMGs, particularly in phosphorus and nitrogen cycling, points to the potential for viral-encoded functions to support host metabolic pathways and reflect adaptation to prevailing environmental conditions. This putative host-virus metabolic linkage is especially significant in nutrient-limited or anthropogenically influenced environments, where such complementary capabilities could potentially provide mutual competitive advantages, ultimately shaping the distinct community patterns observed across the PRE.

## Conclusions

Our comprehensive analysis of the PRE reveals how environmental gradients orchestrate complex interactions between microbial communities, their associated viruses, and biogeochemical processes. By applying an integrated metagenomic framework to both viral and microbial communities, we identified distinct ecological patterns that underscore the estuary’s role as a dynamic transition zone between freshwater and marine ecosystems. The identification of estuarine and oceanic envirotypes, driven primarily by salinity and nutrient availability, highlights sophisticated community adaptations. While the existence of such envirotypes is a known ecological principle, our work provides a more complete picture by simultaneously characterizing the prokaryotic, eukaryotic, and viral contributors to these assemblages. The shift from bacterial-dominated estuarine communities to mixed bacterial-archaeal assemblages in offshore waters mirrors global ocean trends [81] while exhibiting unique characteristics specific to the PRE. Notably, estuarine communities demonstrated enhanced phosphorus cycling pathways and specialized nitrogen metabolism genes, reflecting adaptations to local nutrient dynamics.

Our integrated viral analyses yielded key novel insights into the complexity of virus-host interactions across the estuarine gradient. The discovery of an unexpectedly high abundance of Nucleocytoplasmic Large DNA Viruses (NCLDVs; 44.86%–46.63%) in offshore waters, coupled with elevated levels of their eukaryotic hosts, highlights the tight coupling between viral and host communities. This finding challenges the traditional paradigm of bacteriophage dominance in marine systems and suggests that eukaryotic viruses may play a more significant role in coastal ecosystems than previously acknowledged. The extensive catalog of viral AMGs linked to nutrient cycling provides mechanistic insights into how viruses influence host metabolism and, consequently, ecosystem processes. Phosphorus-related AMGs (*phoH*, *pstS*) and nitrogen metabolism genes (*amoC*, *glnA*) identified in viral genomes mirror their hosts’ metabolic capabilities, suggesting a coordinated adaptation to nutrient availability across biological domains. Such viral-host metabolic synergy is particularly critical in anthropogenically influenced coastal systems like the PRE, where nutrient dynamics are increasingly modified by human activities.

These findings have important implications for understanding how coastal ecosystems respond to environmental change. The clear connections between environmental gradients, community composition, and metabolic capabilities offer a framework for predicting how microbial communities might respond to future perturbations. Given the growing pressures on coastal ecosystems from climate change and anthropogenic activities [82], such predictive understanding is essential for effective ecosystem management and conservation. Looking ahead, our study highlights several critical areas for future research that stem directly from our novel findings. The high proportion of unclassified viral sequences and the potential for cross-phylum infections suggest substantial unexplored viral diversity. Moreover, the elevated levels of xenobiotic biodegradation-related AMGs point to microbial adaptations to anthropogenic influences that warrant further investigation. These observations underscore the need for continued exploration of viral and microbial adaptations in human-impacted estuarine ecosystems.

In conclusion, this study establishes the PRE as a model system for understanding how environmental gradients shape microbial and viral ecology. The novelty of this work lies in the integrated approach we employed, combining environmental, metagenomic, and functional analyses to provide a robust framework for future studies. As these critical transition zones continue to experience rapid environmental change, a detailed understanding of their microbial ecology will be essential for predicting and managing their responses to future perturbations. The intricate interactions we observed between environmental conditions, microbial communities, and viral populations emphasize the importance of a multi-domain perspective when studying coastal ecosystems. Such holistic understanding will be crucial for developing effective management strategies to protect and sustain these vital environments in the face of ongoing global change.

## Supplementary Material

supplementary_pic_20250912_ycaf164

supplementary_tables_20250912_ycaf164

## Data Availability

The raw metagenomic reads analyzed in the current study are available in the NCBI database under the BioProject accession number PRJNA1212835.
