# Supplementary material for: Environmental gradients shape viral-host dynamics in the Pearl River estuary
Source: ISME Commun. 2025 Sep 17;5(1):ycaf164. doi: 10.1093/ismeco/ycaf164 (PMC12533691; doi:10.1093/ismeco/ycaf164)
Supplement: supplementary_pic_20250912_ycaf164 [file supplementary_pic_20250912_ycaf164.pdf]

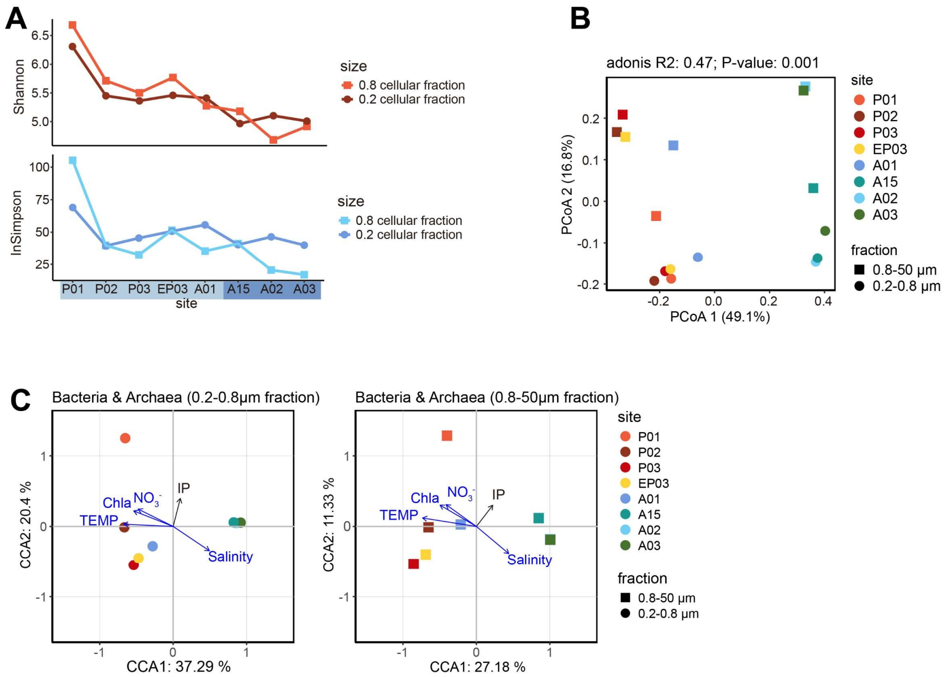

Fig. S1 **Biodiversity of prokaryotic communities across the Pearl River Estuary. A)** Alpha diversity (Shannon and Inverse Simpson indices) of the prokaryotic communities at 8 sites for both size fractions (0.8-50µm, 0.2-0.8µm) based on normalized read counts. **B)** PCoA ordination showing significant clustering of prokaryotic communities by water type (estuarine vs. oceanic; adonis R^2=0.42, P<0.05). **C)** CCA analysis illustrating correlation between prokaryotic community structures and environmental factors (temperature, chlorophyll-a, salinity, inorganic phosphorus (IP), nitrate NO_3^-). Blue vectors represent significant environmental factors (p<0.05).

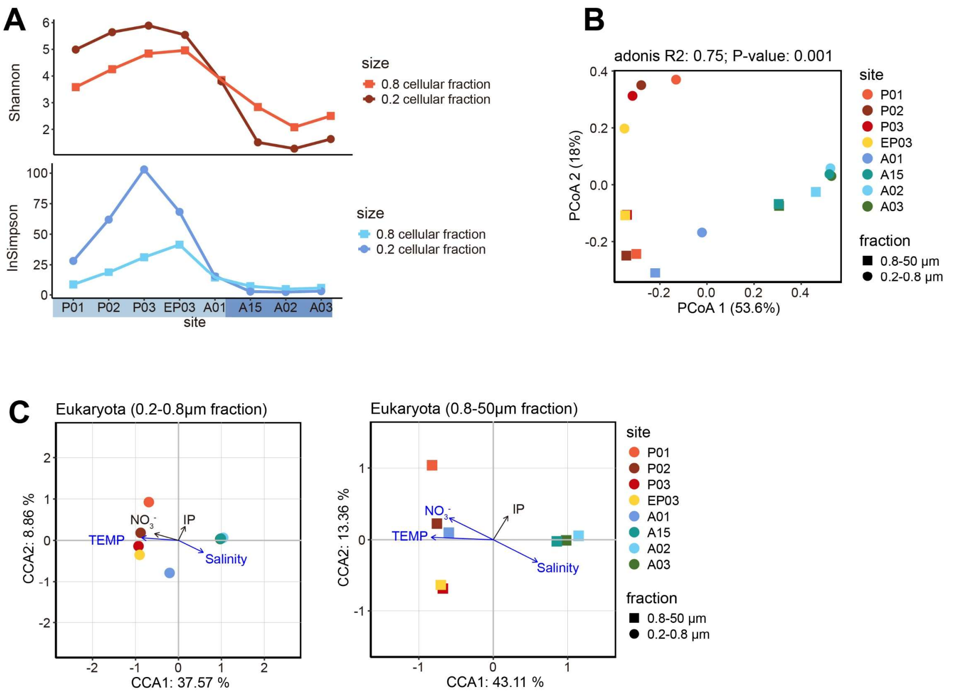

Fig. S2 **Biodiversity of eukaryotic communities sampled in the Pearl River Estuary. A)** Alpha diversity (Shannon and Inverse Simpson indices) of the eukaryotic communities at 8 sites for both size fractions (0.8-50μm, 0.2-0.8μm) based on normalized read counts. **B)** PCoA ordination showing significant clustering of eukaryotic communities by water type (estuarine vs. oceanic; adonis R^2=0.42, P<0.05). **C)** CCA analysis illustrating correlation between eukaryotic community structures and environmental factors (temperature, chlorophyll-a, salinity, inorganic phosphorus (IP), nitrate NO_3^-). Blue vectors represent significant environmental factors (p<0.05).

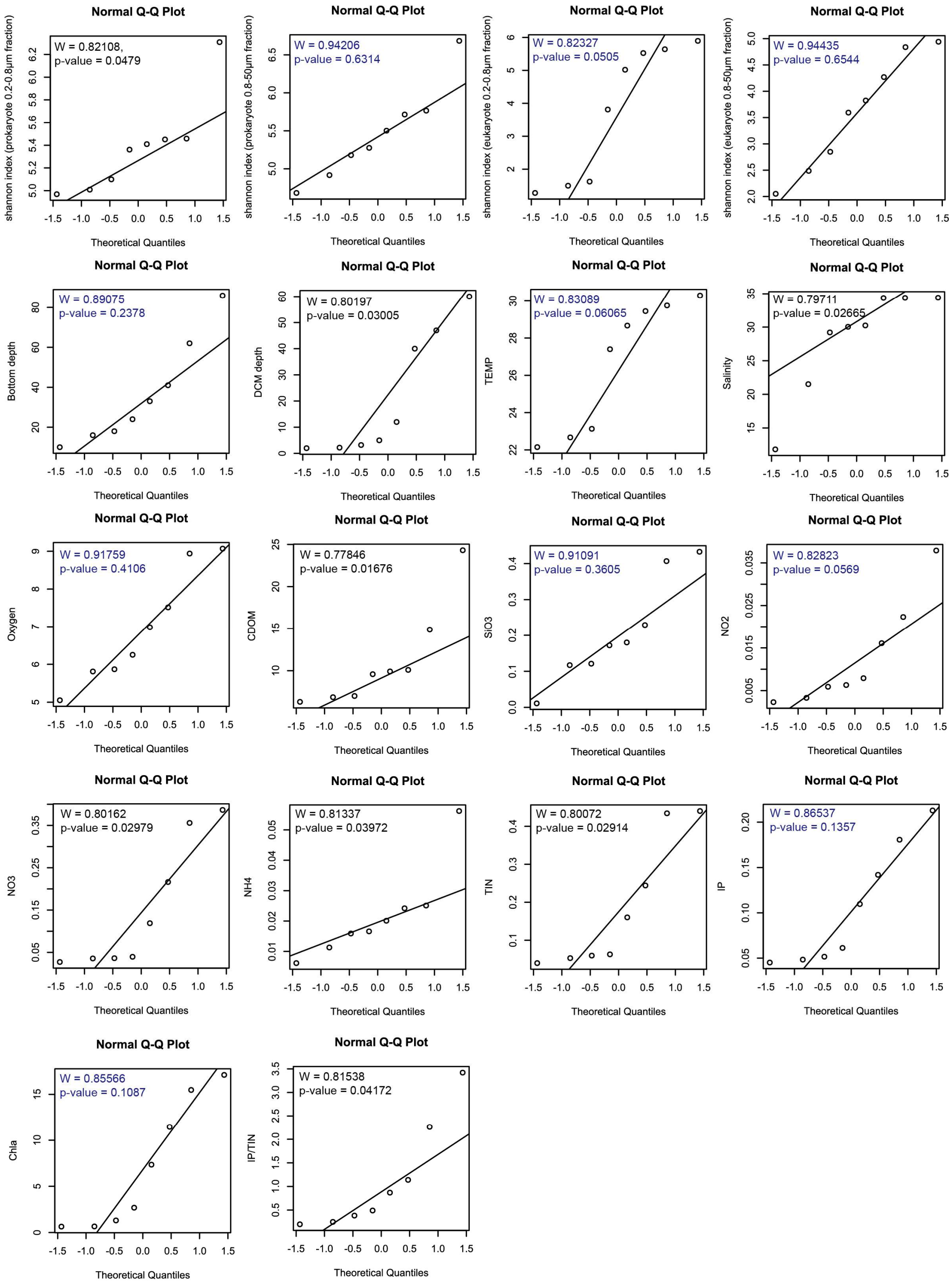

Fig. S3 **Normality was assessed by Q–Q plots coupled with Shapiro–Wilk tests**: W statistics and p-values are annotated, and variables conforming to normality ($p > 0.05$) are highlighted in blue.

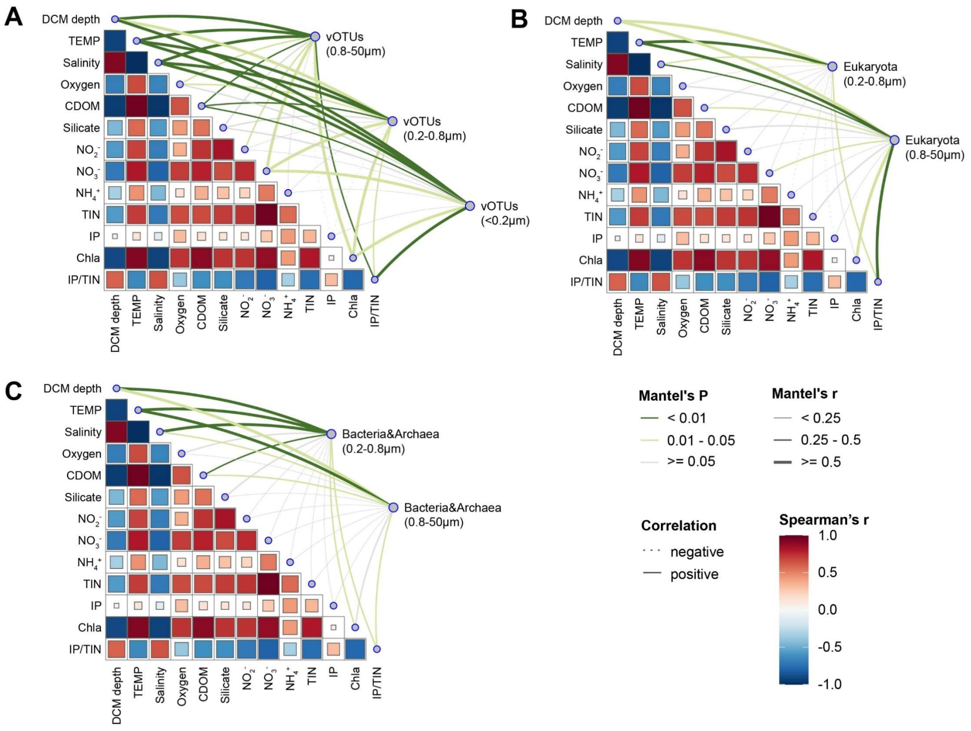

Fig. S4 **Relationships between environmental factors and virus/host communities. (A)** viral communities across three size fractions (<0.2 µm, 0.2–0.8 µm, and 0.8–50 µm). **(B)** Correlations between environmental factors and eukaryotic communities. **(C)** Correlations between environmental factors and bacterial and archaeal communities. Heatmaps display Spearman's correlation coefficients among environmental variables, while lines represent Mantel test (based on Spearman's correlation) results. Line thickness indicates correlation strength (Mantel's *r*), and color denotes statistical significance: dark green ($p < 0.01$), light green ($0.01 \leq p < 0.05$), and light gray ($p \geq 0.05$). Environmental parameters include temperature (TEMP), salinity, dissolved oxygen, and colored dissolved organic matter (CDOM), nutrients (Silicate, NO_3^-, NO_2^-, NH_4^+, TIN, IP), chlorophyll *a* (Chla), and the IP/TIN ratio.

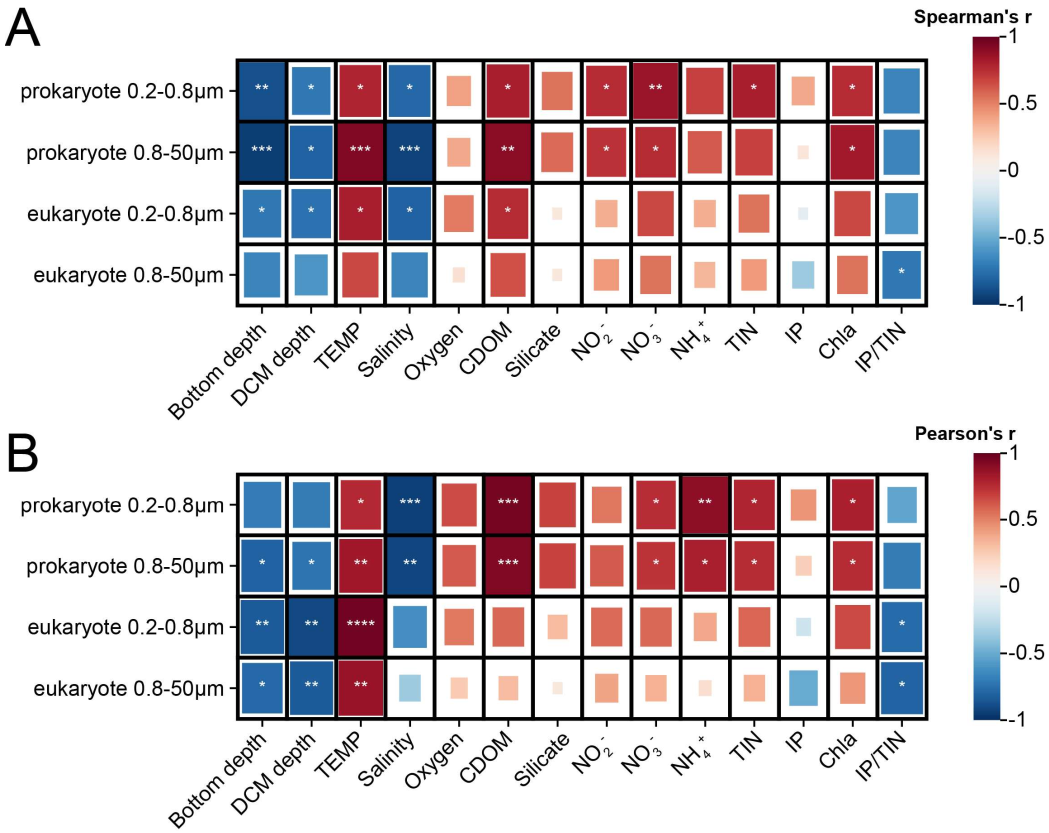

Fig. S5 **The relationship of environmental factors and biodiversity of microbial community.** This heatmap shows the correlation between Shannon indexes of prokaryotic and eukaryotic communities in 0.2-0.8, 0.8-50μm fraction (rows) and environmental factors (columns) based on Pearson correlation **(A)** and Spearman correlation **(B)** Key factors include sampling depth, DCM depth (deep chlorophyll maximum), TEMP (temperature), salinity, CDOM (colored dissolved organic matter), silicate, NO_2^-, NO_3^-, NH_4^+, TIN (total inorganic nitrogen), IP (inorganic phosphorus), Chlorophyll-a, and IP/TN (inorganic phosphorus divided by total nitrogen). The color and size of the squares represent the correlation, with significance levels indicated by asterisks: * $p < 0.05$, ** $p < 0.01$, and *** $p < 0.001$.

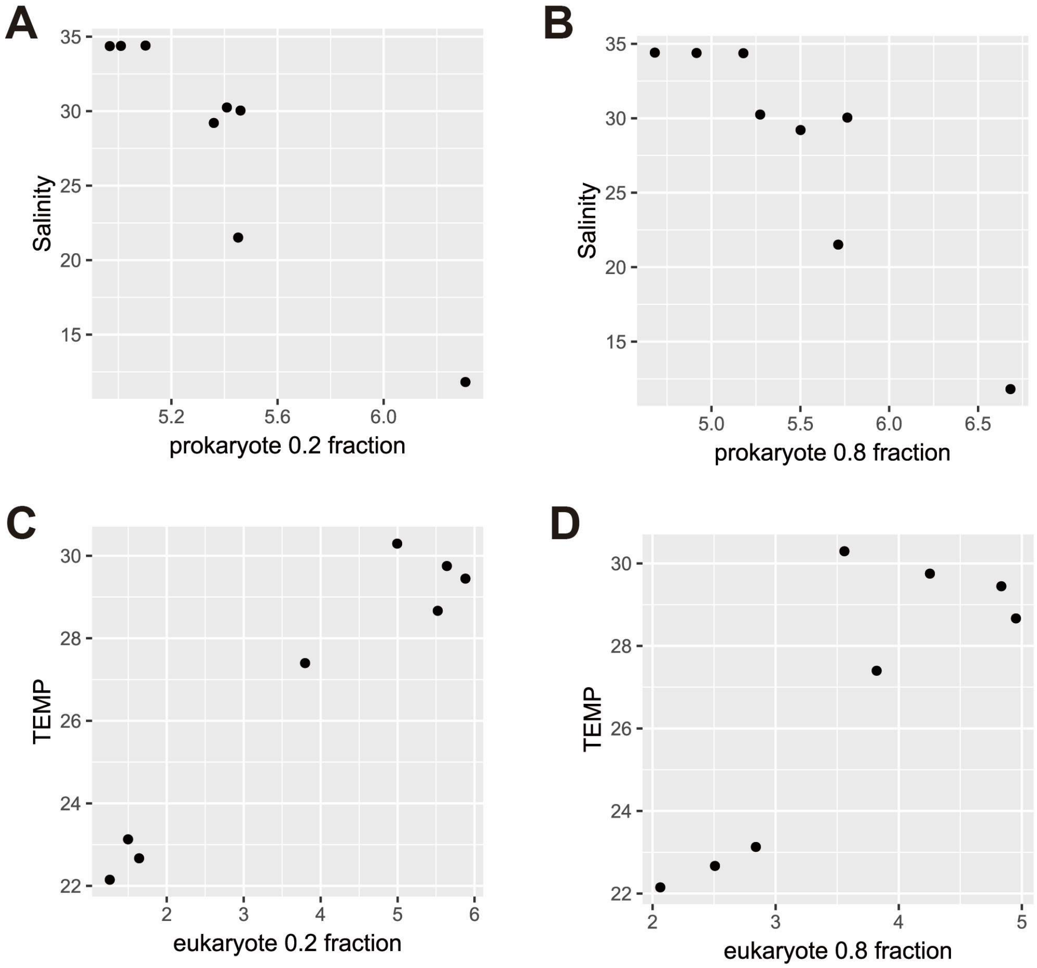

Fig. S6 **The scatter plots of Shannon diversity against key environmental factors (salinity and temperature) are presented as follows**: **A)** and **B)** show the relationship between the diversity of the prokaryotic community (0.2-0.8 μm and 0.8-50 μm) and salinity, while **C)** and **D)** illustrate the relationship between the diversity of the eukaryotic community (0.2-0.8 μm and 0.8-50 μm) and temperature.

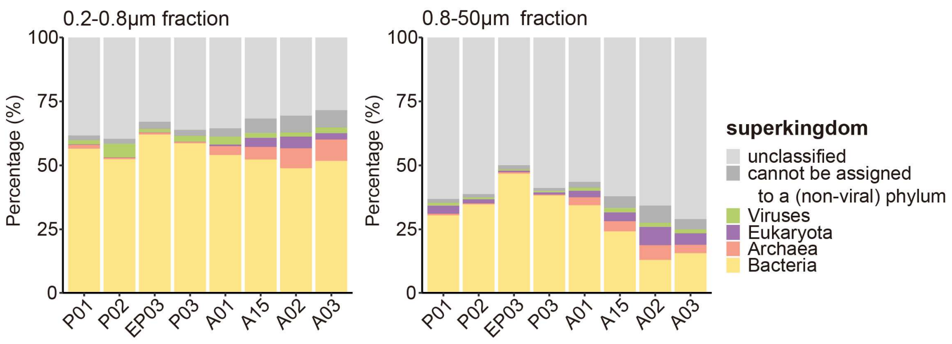

Fig. S7 **Superkingdom-level classification of metagenomic reads.** Relative abundance of reads assigned to Bacteria, Archaea, Eukaryota, Viruses, and unclassified sequences in 0.2-0.8μm fraction and 0.8-50μm size fractions. Taxonomic assignments were performed using Kaiju.

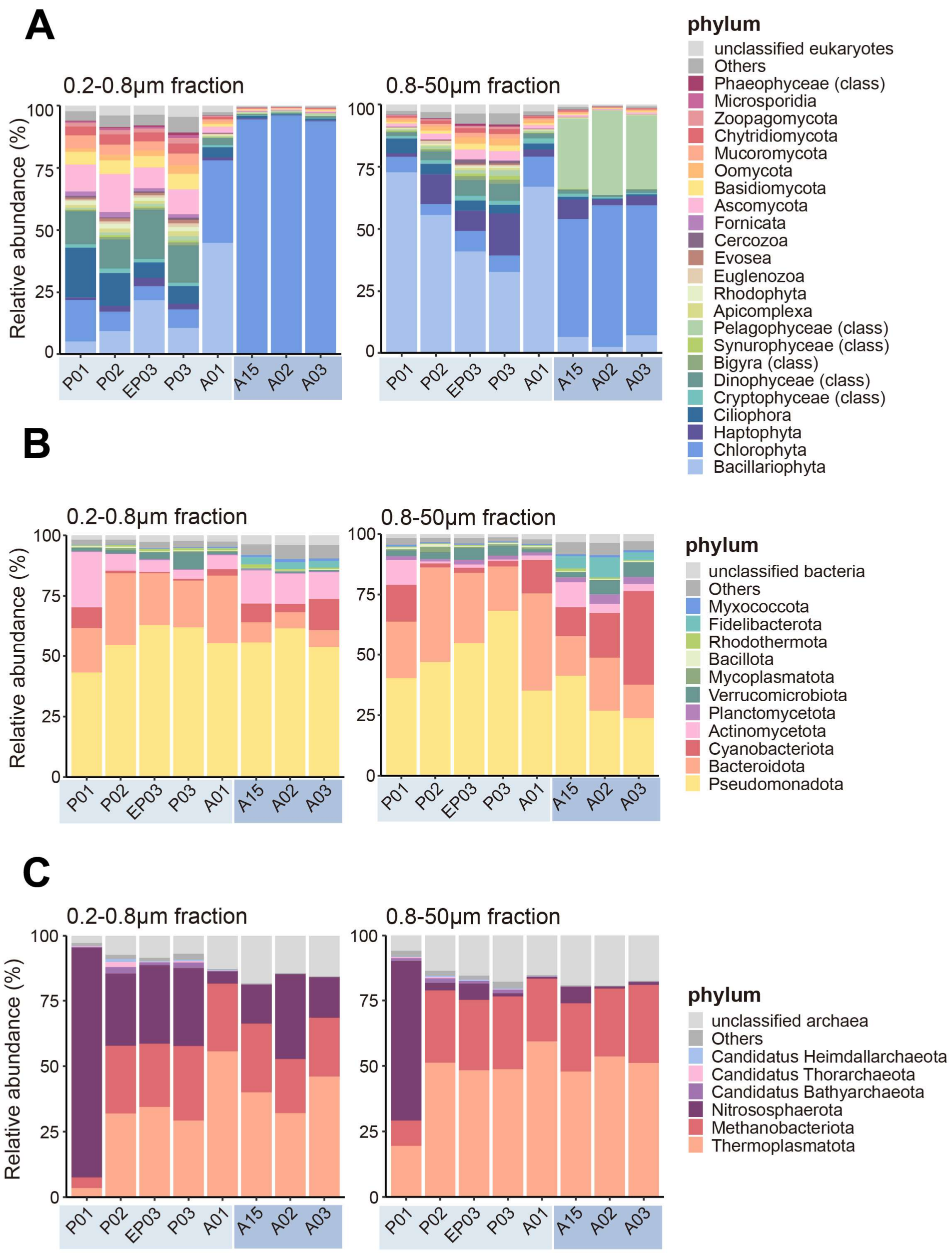

Fig. S8 **Taxonomy profiles of microbial communities based on sequencing reads.** **A)** Eukaryotic communities, **B)** Bacterial communities, and **C)** Archaeal communities in two size fractions: 0.2-0.8μm and 0.8-50μm. Taxa with less than 1% relative abundances across all samples are grouped as 'Others'.

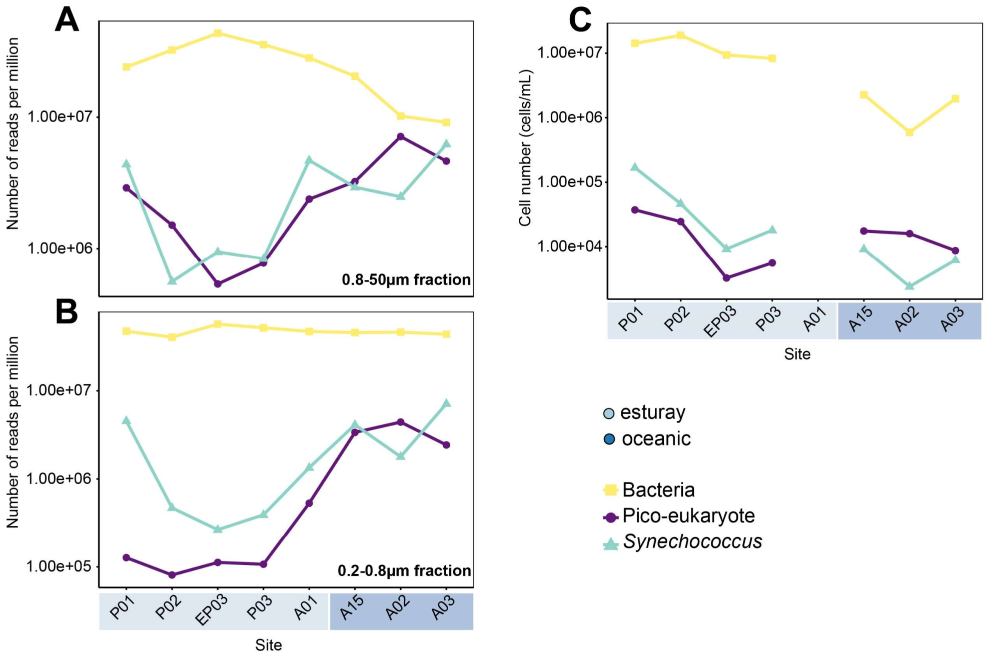

Fig. S9 **The abundances of different microbe groups.** Metagenomic read-based abundance of bacteria in the 0.8-50 µm **(A)** and 0.2-0.8 µm **(B)** size fractions, expressed as reads per million. Taxonomic classification was performed using Kaiju. **C)** Absolute cell abundances (cells/mL) of the same microbial groups determined by flow cytometry. Flow cytometry data for station A01 was not collected.

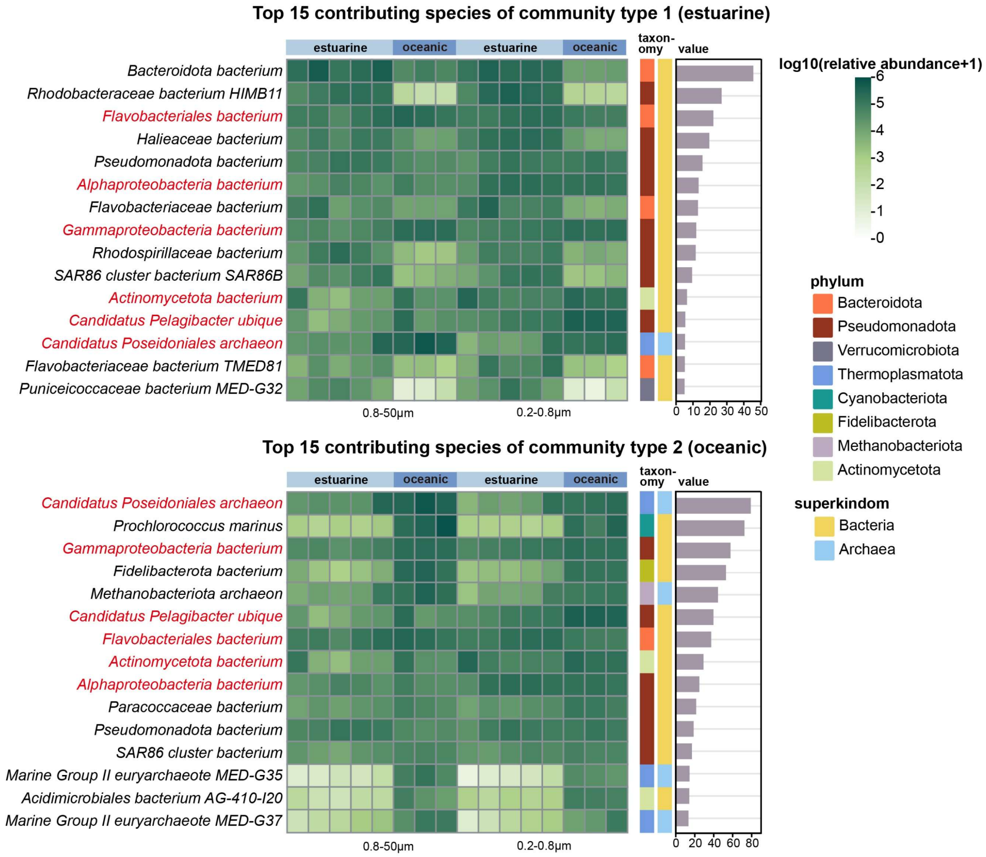

Fig. S10 **Dirichlet multinomial mixtures (DMM) analysis of prokaryotic communities.** DMM clustering identified two distinct community types: estuarine (top panel) and oceanic (bottom panel), showing the top 15 contributing species for each community type. The heatmap displays log-transformed relative abundance across the eight sampling sites arranged from estuarine to oceanic environments. Taxonomic classification is indicated by color bars showing phylum and superkingdom affiliations. The rightmost bar charts quantify each species' contribution value to its respective community type.

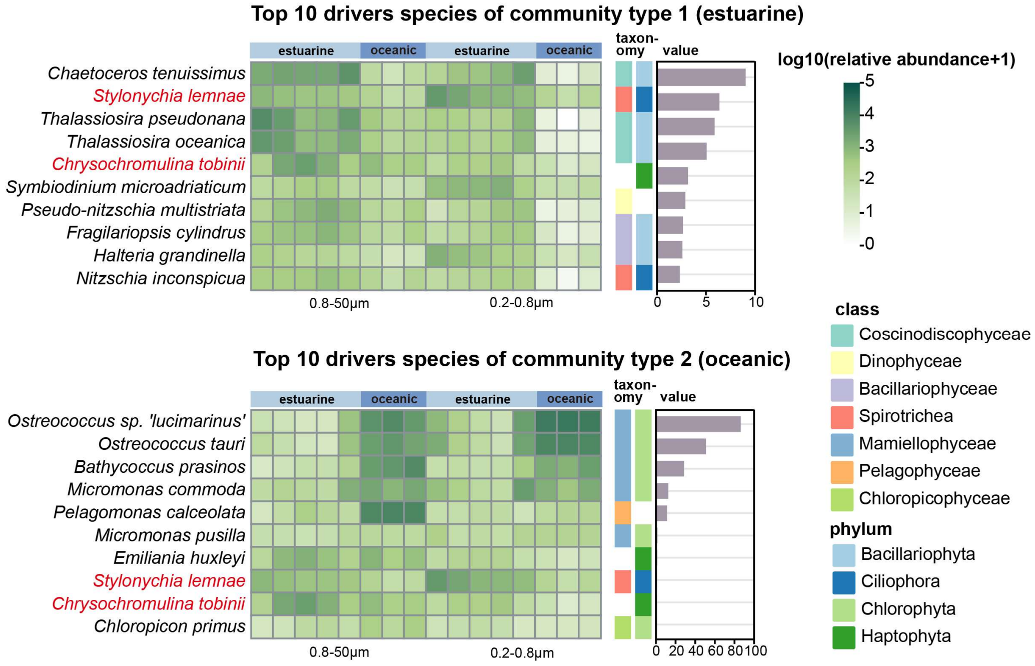

Fig. S11 **Dirichlet multinomial mixtures (DMM) analysis of eukaryotic communities.** DMM clustering identified two distinct community types: estuarine (top panel) and oceanic (bottom panel), showing the top 10 contributing species for each community type. The heatmap displays log-transformed relative abundance across the eight sampling sites arranged from estuarine to oceanic environments. Taxonomic classification is indicated by color bars showing phylum and class affiliations. The rightmost bar charts quantify each species' contribution value to its respective community type.

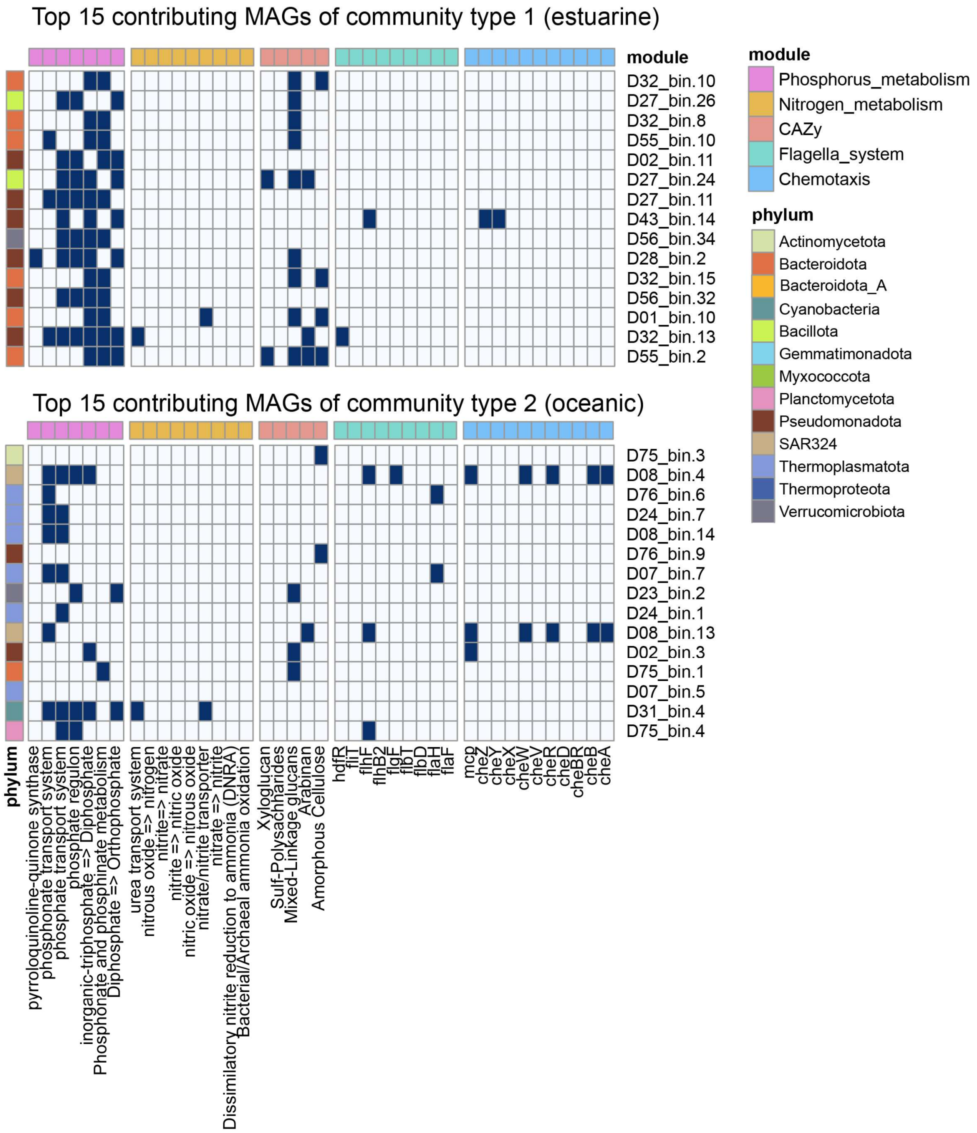

Fig. S12 **Metabolic profile of the top 15 estuarine and oceanic ecotypes.** The heatmap depicts the presence (blue) or absence (white) of key functional genes involved in biogeochemical cycling processes and environmental adaptation. Genes are grouped into functional modules (color-coded at top): phosphorus metabolism, nitrogen metabolism, carbon fixation (CAZy), flagella system, and chemotaxis. Each row represents one of the top 15 most abundant MAGs from either estuarine (top panel) or oceanic (bottom panel) communities as determined by DMM analysis. The color bar on the left indicates phylum-level taxonomy.

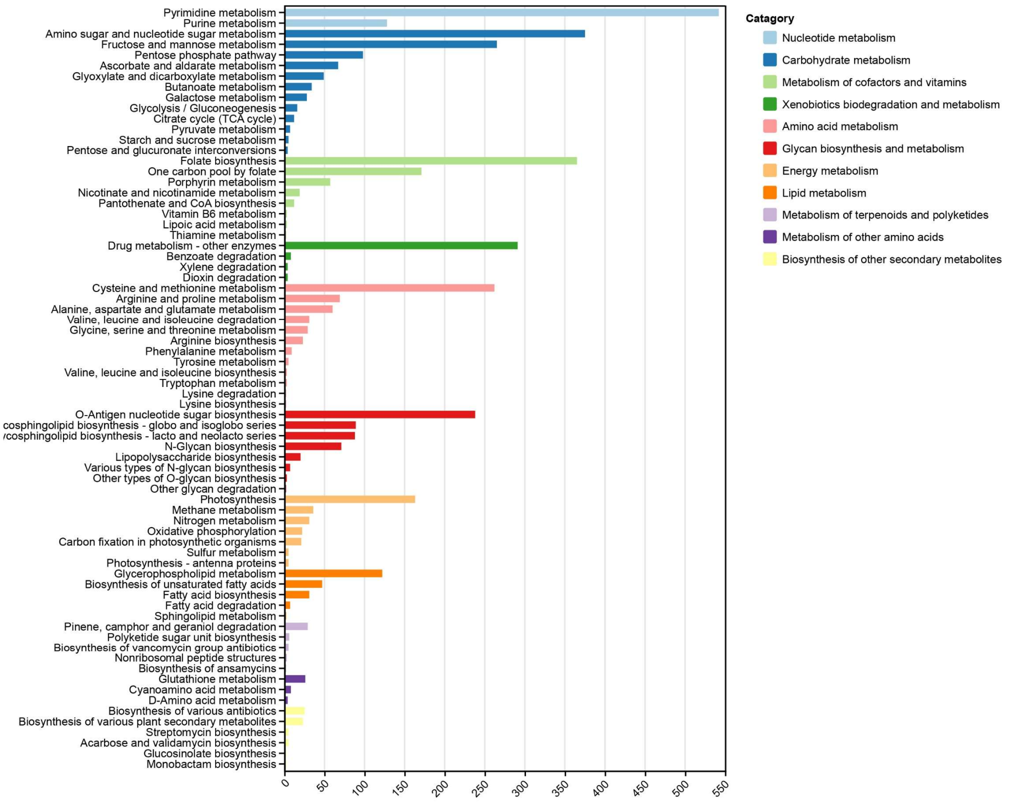

Fig. S13 **Auxiliary Metabolic Genes (AMGs) in Viral Genomes Involved in Metabolism.** This bar plot illustrates the counts of AMGs identified in viral genomes annotated by the KEGG database, organized by metabolic pathways (in colors). Only pathways with more than one associated AMG are displayed in the graph.
